# A Novel Polysaccharide Depolymerase Encoded by the Phage SH-KP152226 Confers Specific Activity Against Multidrug-Resistant *Klebsiella pneumoniae via* Biofilm Degradation

**DOI:** 10.3389/fmicb.2019.02768

**Published:** 2019-12-03

**Authors:** Yunqiang Wu, Rui Wang, Mengsha Xu, Yanan Liu, Xianchao Zhu, Jiangfeng Qiu, Qiming Liu, Ping He, Qingtian Li

**Affiliations:** ^1^Department of Laboratory Medicine, Ruijin Hospital, Shanghai Jiao Tong University School of Medicine, Shanghai, China; ^2^Department of Research, Renji Hospital, Shanghai Jiao Tong University School of Medicine, Shanghai, China; ^3^Department of Immunology and Microbiology, Shanghai Jiao Tong University School of Medicine, Shanghai, China; ^4^Department of Gastrointestinal Surgery, Shanghai Ruizhou Biotech Co. Ltd., Shanghai, China

**Keywords:** carbapenem-resistant *Klebsiella pneumoniae*, bacteriophage, depolymerase, biofilm, capsular polysaccharide

## Abstract

The increasing prevalence of infections caused by multidrug-resistant *Klebsiella pneumoniae* necessitates the development of alternative therapies. Here, we isolated, characterized, and sequenced a *K. pneumoniae* bacteriophage (SH-KP152226) that specifically infects and lyses *K. pneumoniae* capsular type K47. The phage SH-KP152226 contains a genome of 41,420 bp that encodes 48 predicted proteins. Among these proteins, Dep42, the gene product of ORF42, is a putative tail fiber protein and hypothetically possesses depolymerase activity. We demonstrated that recombinant Dep42 showed specific enzymatic activities in the depolymerization of the K47 capsule of *K. pneumoniae* and was able to significantly inhibit biofilm formation and/or degrade formed biofilms. We also showed that Dep42 could enhance polymyxin activity against *K. pneumoniae* biofilms when used in combination with antibiotics. These results suggest that combination of the identified novel depolymerase Dep42, encoded by the phage SH-KP152226, with antibiotics may represent a promising strategy to combat infections caused by drug-resistant and biofilm-forming *K. pneumoniae*.

## Introduction

*Klebsiella pneumoniae* is an opportunistic bacterial pathogen that causes a wide range of nosocomial infections. It widely colonizes the human mouth, skin, respiratory tract, and digestive tract under normal physiological conditions. *K. pneumoniae* can become pathogenic in patients with compromised immune systems, causing various diseases such as urinary tract infections, pyogenic liver abscess (PLA), and pneumonia (Podschun and Ullmann, 1998; Hsieh et al., 2008). The increasing prevalence of carbapenem-resistant *Klebsiella pneumoniae* (CRKP) has led to the loss of the effectiveness of antibiotics against such infections (Keynan and Rubinstein, 2007; Coque et al., 2008; Munoz-Price et al., 2013). Thus, the Centers for Disease Control (CDC) of the USA designated *K. pneumoniae* as an urgent threat to public health (Solomon and Oliver, 2014).

One of the crucial pathogenic factors of *K. pneumoniae* is the capsular polysaccharide (CPS), which either exists on the surface of the bacterial cells or is released to the surrounding environment as an exopolysaccharide. The CPS is typically composed of tens to thousands of repeating units consisting of several monosaccharides, and *in vivo* synthesis of CPS is controlled by the CPS synthesis locus in *K. pneumoniae* (Podschun and Ullmann, 1998). There have been at least 78 capsular serotypes of *K. pneumoniae* documented, the CPS structures of which vary significantly (Pan et al., 2008, 2013; Majkowska-Skrobek et al., 2016). The CPS on the bacterial surface not only plays a crucial role in the evasion of the host defense system by bacteria (Li et al., 2014) but also serves as a significant barrier to effective penetration by antibiotics (Stewart, 1996; Samson et al., 2013). In addition, the extracellular polysaccharide (EPS) released by *K. pneumoniae* may contribute to biofilm formation (Li et al., 2014), which confers high tolerance against antimicrobial agents to the bacteria and potentially leads to severe persistent infections (Anderl et al., 2000; Dowd et al., 2008). Biofilm formation has been reported to be associated with many medical conditions, such as urinary tract infections, endocarditis, peritonitis *via* indwelling medical devices, and dental plaque (Hassan et al., 2011). It is estimated that more than 65% of all bacterial infections involve biofilms (Römling and Balsalobre, 2012; González et al., 2018), and the most common bacterial biofilms that cause device-related infections are *K. pneumoniae* biofilms (Singhai et al., 2012). Due to the inherent tolerance of biofilms to antimicrobial agents, there is a growing need for the development of alternate strategies to control biofilm-associated infections (Ansari et al., 2014).

As bacterial predators, bacteriophages have evolved various virion-associated depolymerases that can depolymerize the polysaccharides involved in CPS and biofilm formation and facilitate the access of the bacteriophage to the bacterial surfaces (Leiman et al., 2007; Leiman and Molineux, 2008; Li et al., 2016). The hallmark characteristic of the existence of phage-encoded polysaccharide depolymerases is plaque-surrounding halos, which grow in diameter with increasing incubation time (Cornelissen et al., 2012; Harper et al., 2014; Pires et al., 2016). Phages with depolymerase activity can degrade polysaccharides such as CPS and EPS and thus remove the physical barrier for access to host receptors (Adams and Park, 1956; Drulis-Kawa et al., 2015). In addition, depolymerization of CPS or EPS makes encapsulated bacteria devoid of an important shield and more susceptible to the host immune defense system as well as antimicrobial agents (Mushtaq et al., 2004, 2005; Bansal et al., 2014). In previously published studies, various depolymerases used as adjuvants have been reported to fight against antimicrobial-resistant bacteria by preventing biofilm formation or degrading existing biofilms. Lu and Collins found that a bacteriophage engineered to express a depolymerase enzyme can degrade *Escherichia coli* biofilms to a significant degree compared with the nonengineered control bacteriophage (Lu and Collins, 2007). Bansal and his colleagues showed that a depolymerase encoded by *Aeromonas punctata* was able to degrade the capsule of *K. pneumoniae*, which resulted in restored sensitivity to gentamicin (GEN) for antibacterial treatment (Bansal et al., 2015). These data suggest that the use of phage depolymerases is an attractive approach for the treatment of biofilm-associated infections.

To date, various depolymerases have been identified and reported for 20 different capsular types of *K. pneumoniae* (Pan et al., 2019). However, no report has been published on a depolymerase that specifically targets K47-type *K. pneumoniae*. In this study, we first report the isolation and characterization of a novel lytic phage, SH-KP152226, and a depolymerase (Dep42) against K47-type *K. pneumoniae*. Our data showed that Dep42 could specifically degrade the CPS of *K. pneumoniae* (strain 2226). We also investigated the potential application of this depolymerase in the control of multidrug-resistant bacteria through biofilm degradation.

## Materials and Methods

### Bacterial Strain Isolation and Identification

Sixteen *K. pneumoniae* clinical isolates were obtained from Huashan Hospital, affiliated with Fudan University, between 2015 and 2016. The clinical isolates were identified with the Vitek 2 compact system (Biomerieux, France). The antibiotic sensitivity was determined using the Kirby-Bauer (K-B) method. Evaluation of the capsular type was carried out by *wzi* sequencing according to the method of Brisse S (Brisse et al., 2013). All bacterial strains were stored frozen at −80°C in 50% (v/v) glycerol and were routinely grown in lysogeny broth (LB, Sangon Biotech, China) at 37°C. The viable bacterial count was determined using LB agar (1.5% w/v) plates. Tryptic soy broth (TSB) medium (Sangon Biotech, China) was used to culture bacterial biofilms.

### Multilocus Sequence Typing and Pulsed-Field Gel Electrophoresis

Multilocus sequence typing (MLST) was performed on isolates according to the protocol described on the *K. pneumoniae* MLST website^1^. MLST results were analyzed using the international *K. pneumoniae* MLST database created in 2005 at the Institut Pasteur (Diancourt et al., 2005). Pulsed-field gel electrophoresis (PFGE) typing of eight ST11 isolates (*K. pneumoniae* strains 1044, 1093, 1115, 2226, 2302, 2340, 2450, and 2482) was performed as previously reported (Ribot et al., 2006). DNA fingerprints were obtained from PFGE profiles of genomic DNA digested with *Xba*I (New England Biolabs, USA).

### Bacteriophage Isolation

Sewage samples were obtained from Huashan Hospital, affiliated with Fudan University. Briefly, the water sample was centrifuged, enriched with *K. pneumoniae* strain 2226, and incubated under shaking conditions at 37°C overnight. The supernatant containing the phages was collected, filtered using a sterile 0.22-μm filter (Millex-GP Filter Unit; Millipore, USA), serially diluted in SM buffer (100 mM NaCl, 8 mM MgSO_4_ 7H_2_O, 50 mM Tris-HCl, pH 7.5), and spotted on a double-layer agar plate of *K. pneumoniae* strain 2226 for phage detection and isolation. Phage plaques were purified continuously until the plate presented a single plaque morphology. Purified phage plaques were amplified in plates as described previously (Pires et al., 2011) with minor modifications. Three milliliters of SM buffer was added to the plates, and the plates were incubated under agitation (120 rpm) at 4°C for 4 h. Then, the liquid and the top agar were collected and centrifuged at 9000×*g* for 15 min; the supernatant was removed and filtered with 0.22-μm filters. One lytic phage was obtained and designated as SH-KP152226. The purified phage was stored in SM buffer at 4°C.

### Purification of the Phage SH-KP152226

The phage SH-KP152226 was enriched at 4°C overnight by using polyethylene glycol (PEG) 8000 (Sangon Biotech, China) and further purified by CsCl density gradient (1.33, 1.45, 1.50, and 1.70 g/cm^3^) ultracentrifugation at 4°C and 20,000×*g* for 4 h (Beckman, JA-25.50, USA). Then, the layer containing the enriched phage was collected using a syringe, dialyzed against SM buffer, and stored at 4°C.

### Transmission Electron Microscopy

The purified phage was dropped onto copper grids provided with carbon-coated Formvar films and stained with phosphotungstic acid (2% w/v). A Hitachi transmission electron microscope H-9500 (Japan) was used to observe the morphology of the phage.

### Genomic DNA Sequencing and Annotation

The genomic DNA of SH-KP152226 was extracted by the standard protocol as previously described (Ramos et al., 1995; Guo et al., 2017). Briefly, the concentrated phage was treated with 10 μg/ml DNase I and RNase A (Sigma-Aldrich, USA) in SM buffer at 37°C for 1 h. Subsequently, 25 mmol/ml ethylenediaminetetraacetic acid (EDTA) was added. Finally, phage DNA was extracted using the Qiagen Lambda Kit (Qiagen, USA).

The DNA library was constructed using the ABclonal DNA Library Preparation Kit for Illumina and sequenced (150 bp in paired-end mode) using the Illumina HiSeq 3000 platform (Illumina, USA). A total of 222,265 trimmed reads were obtained. SOAPdenovo2 software was used for genome assembly and optimization (Luo et al., 2015). The open reading frames (ORFs) of the phage genome were predicted using Glimmer 3.02 software and GeneMark (Delcher et al., 1999). Annotation of these predicted genes was conducted by comparison with known sequences using the BLAST online tool from the NCBI website^2^. The results of the HHpred database were used to locate the gene encoding polysaccharide depolymerase (Uniclust was chosen as the multiple sequence alignment (MSA) generation method, and three was chosen as the maximal number of MSA generation steps) (Soding et al., 2005; Johnson et al., 2008).

### Cloning, Expression, and Purification of the Recombinant Depolymerase

The ORF42 gene of the phage SH-KP152226 encoding the depolymerase was amplified from purified SH-KP152226 by PCR using the specific primers F (5′-CGAGCTCATGGACCAAGACATTAAAACAGTC-3′) and R (5′-CCCAAGCTTTTACTGTTCGCCCCACTGC-3′), which introduced *Sac* I and *Hind* III restriction sites on each side of the amplified fragment. The PCR product was digested with *Sac* I and *Hind* III (New England Biolabs) and cloned into the pSUMO3 expression vector (LifeSensors, USA). The recombinant plasmid was transformed into *E. coli* BL21 (DE3) competent cells. The BL21 (DE3) cells harboring the recombinant plasmid were grown in 1 L of LB supplemented with 50 μg/ml ampicillin at 37°C under shaking conditions to an optical density (OD_600_) of ~0.6. The recombinant protein was induced with 0.5 mM isopropyl-β-D-thiogalactopyranoside (IPTG, Sangon Biotech, China), followed by shaking at 30°C for 4 h. The culture was harvested by centrifugation at 5000×*g* and 4°C for 30 min (Beckman, JA-8.10, USA), and the cells were collected and resuspended in 20 ml of lysis buffer (50 mM Tris-HCl, 500 mM NaCl, 10% glycerol, 20 mM imidazole, pH 7.5) containing phenylmethylsulfonyl fluoride (PMSF, Beyotime Biotech, China). The resuspended cells were lysed on ice using a high-pressure homogenizer (JNBIO, China). The cell debris was removed by centrifugation at 12,000×*g* and 4°C for 1 h (Beckman, JA-25.50, USA). The SUMO-His-tagged protein was purified using nickel-nitrilotriacetic acid (Ni-NTA) resin (GE Healthcare, USA) in a gravity column according to the manufacturer’s instructions. Briefly, the column was equilibrated with lysis buffer (50 mM Tris-HCl, 500 mM NaCl, 10% glycerol, 20 mM imidazole, pH 7.5); the supernatant was loaded into the column, followed by washing with 20 volumes of lysis buffer. The fusion protein was eluted with elution buffer (20 mM Tris-HCl, 50 mM NaCl, 300 mM imidazole, pH 7.5).

The purified recombinant depolymerase protein (designated as Dep42) labeled with His-SUMO Tag was concentrated using ultrafiltration. SUMO protease (LifeSensors, USA) was added to the solution at a ratio of 1 unit of enzyme per 100 μg of substrate. The solution was dialyzed in dialysis buffer (50 mM Tris-HCl, 50 mM NaCl, 10% glycerol, 20 mM imidazole, pH 7.5) at 4°C overnight. The digested protein solution was then reapplied to the nickel column to remove the His-tagged SUMO and undigested fusion proteins, and the recombinant protein without the tag was collected in the flow-through. Fractions from each purification step were analyzed in sodium dodecyl sulfate-polyacrylamide gel electrophoresis (SDS-PAGE) gels stained with Coomassie blue (Thermo Scientific, USA). A molecular weight (MW) standard (10–170 kDa) (Thermo Scientific, USA) was used for SDS-PAGE. The purified protein was concentrated by ultracentrifugation using a 30-kDa MW cut-off (MWCO) membrane (Thermo Scientific, USA) and stored at −80°C.

### Depolymerization Activity Assay

The double-layer agar plate method was used to determine the host range of the phage SH-KP152226 and the activity of recombinant Dep42 against various serotypes of *K. pneumoniae* (Table 1). Log-phase bacterial cultures of *K. pneumoniae* were poured onto an LB soft agar overlay plate (LB with 0.7% agar) to form lawns. After drying, 5 μl of the phage suspension (10^8^ PFU/ml) or Dep42 (10 μg/ml) was spotted on the bacterial lawn, and the plate was incubated at 37°C overnight. The formation of lytic zones (plaque) or clear zones (halo) on the bacterial lawn was an indication of the antibacterial activity of the phage or Dep42. Dep42 activity was assessed using the spot assay on *K. pneumoniae* strain 2226 and varying the enzyme concentrations from 5 mg/ml to 0.5 μg/ml. The SUMO protein at 5 mg/ml was used as a negative control.

**Table 1:** The phage and Dep42 spectra for the clinical isolates of *K. pneumoniae* used in this study.

Isolate no.	Capsular type	ST	SH-KP152226		EOP	Dep42
			Plaques	Halos		
7	10	76	−	−	−	−
10	38	278	−	−	−	−
20	3	687	−	−	−	−
21	38	278	−	−	−	−
29	54	180	−	−	−	−
35	2	NA	−	−	−	−
1025	38	11	−	−	−	−
1044	64	11	−	−	−	−
1093	47	11	+	+	+(EOP = 0.77)	+
1115	47	11	+	+	+(EOP = 0.44)	+
2226	47	11	+	+	+(EOP = 1.00)	+
2268	N2	105	−	−	−	−
2302	47	11	+	+	+(EOP = 1.32)	+
2340	47	11	+	+	+(EOP = 0.93)	+
2450	64	11	−	−	−	−
2482	64	11	−	−	−	−

*The capsular type of all *K. pneumoniae* isolates was determined by *wzi* sequencing. The sequence type (ST) was evaluated using the multilocus sequence analysis tool. The phage and Dep42 spectra were tested on all 16 *K. pneumoniae* isolates using drop tests (−, no lysis; +, showed plaques or halos). For the phage, EOP was calculated as the ratio of the number of lysis plaques produced on the lawn of the host bacterium to the number of plaques produced on the lawn of the host strain. NA, sequence type is not available*.

To quantitate the lysis ability of the phage, the efficiency-of-plating (EOP) assay was conducted on different hosts as previously described with some modifications (Hernandez-Morales et al., 2018). In brief, phage samples with a series of 10-fold dilutions were prepared with SM buffer. For plating, 100 μl of *K. pneumoniae* cultures grown to the exponential phase were mixed with 10 μl of the phage suspension; the mixtures were incubated for 10 min, and 4 ml of 0.7% top agar was added. The mixture was then poured on the bottom agar, and the plate was incubated at 37°C for 18 h. The EOP value was calculated as the ratio of the number of lysis plaques produced on the bacterial lawn of the target to the number of plaques produced on the lawn of the host strain. The EOP was recorded as high at a ratio ≥ 0.5 or low at a ratio < 0.5. All experiments were performed in triplicate.

### Purification of the Bacterial Extracellular Polysaccharide

Fresh TSB medium was inoculated with an overnight culture of *K. pneumoniae* strain 2226 and incubated without agitation at 37°C for 5 days. EPS purification was performed as described previously (Bales et al., 2013). Briefly, 60 μl of formaldehyde solution (36.5%) was added to every 10 ml of culture, and the culture was incubated at 100 rpm for an hour. Then, 1 M NaOH was added to the culture, followed by agitation at room temperature for 3 h. Cell suspensions were centrifuged at 16,800×*g* for 1 h (Beckman, JA-25.50, USA), and the supernatant was treated with trichloroacetic acid (TCA, 20% w/v) to remove protein and nucleic acid impurities. The solution was centrifuged at 16,800×*g* for 1 h (Beckman, JA-25.50, USA); the supernatant was collected, and 1.5 volumes of cold ethanol (96%) was added to precipitate the exopolysaccharides at −20°C for 24 h. The precipitate was pelleted by centrifugation at 16,800×*g* for 1 h and resuspended in ddH_2_O. The EPS mixture was dialyzed against an excess of ddH_2_O at 4°C for 24 h using a 12–14 kDa MWCO membrane (Thermo Scientific, USA). The dialyzed EPS was lyophilized.

### Depolymerization of Extracellular Polysaccharide by Dep42

To demonstrate that recombinant the Dep42 had depolymerase activity for the polysaccharide, we performed a depolymerization assay using size exclusion high-performance liquid chromatography (SEC-HPLC). The purified EPS was dissolved in 50 mM Na_2_HPO_4_ (pH 7.0) to a final concentration of 0.5 mg/ml and incubated with Dep42 (10 μg/ml) or SUMO (10 μg/ml) at 37°C for 30 min. After incubation, the reactions were stopped by heating at 100°C for 10 min. Subsequently, the mixture was analyzed by an HPLC instrument (Waters, USA) equipped with a TSKgel G5000 PWxl analytical column (7.8 mm ID × 300 mm) (Tosoh Corporation Bioscience, Japan) and a refractive index detector (Waters, USA). The column was run with sodium phosphate buffer at pH 7.0 as the mobile phase at 1 ml/min and 30°C.

### Determination of Depolymerase Stability

The depolymerization activity of Dep42 was evaluated at pH 3.0–9.0 and 25–90°C by measuring the production of reducing sugars after enzymatic cleavage of the polysaccharide according to the method of Miller with slight modifications (Miller, 1959). Glucose was used as the analytical standard at concentrations ranging from 0.2 to 1 mg/ml. Aliquots of each standard solution (500 μl) were placed in test tubes, and 1.5 ml of DNS was added to each tube. The mixed solutions were then boiled at 100°C for 5 min and diluted to 1:5 (vol/vol) with ddH_2_O after cooling to room temperature. Subsequently, the absorbance at 550 nm was measured with a spectrophotometer, and the obtained values were used to plot the calibration curve. Reducing sugars released after the enzymatic assay were calculated by linear regression as glucose equivalents. The effect of pH on the enzyme activity was tested at 25°C using different buffers: 50 mM sodium acetate buffer (pH 3.0–5.0), 50 mM Na_2_HPO_4_ (pH 6.0–7.0), 50 mM Tris-HCl buffer (pH 8.0–9.0), and 100 mM NaHCO_3_ (pH 10.0–11.0). The effect of temperature was also assessed in 50 mM Na_2_HPO_4_ (pH 6.0) from 25°C to 90°C. The results are presented as the relative activity compared with the best activity value obtained at pH 6.0 and 25°C. To determine the stability under various conditions, the Dep42 protein was first preincubated at different temperatures or pH values for 30 min, and the enzyme activity was measured as described above.

### Biofilm Formation

Biofilm formation was carried out in a 96-well plate as described previously with some modifications (O’Toole and Kolter, 1998). Briefly, *K. pneumoniae* strain 2226 grown overnight at 37°C was diluted to 1:100 in fresh TSB medium, and 200 μl of the diluted culture was added to the well of a plate. The biofilm was allowed to form for 48 h, and 100 μl of the diluted culture was reinoculated into fresh TSB every 24 h. The plates were incubated at 37°C without agitation. Wells with 200 μl of fresh TSB medium were used as the negative controls. After incubation, the supernatant was removed, and the wells were rinsed twice with 200 μl of PBS. Each well was then treated with 200 μl of 10% methanol (v/v) at room temperature for 15 min. The methanol was removed, and the plate was allowed to dry at room temperature. Subsequently, 200 μl of crystal violet (CV, 1% v/v) was added to each well for 20 min, and the wells were washed gently with sterile water. Finally, 100 μl of acetic acid (33% v/v) was added to solubilize the stain. The absorbance was measured at 595 nm using a microplate reader (Thermo Scientific, USA). Each experiment was performed in triplicate.

### Antibiofilm Activity and Antibacterial Activity

The *K. pneumoniae* strain 2226 was used to estimate the ability of Dep42 to inhibit biofilm information. In brief, *K. pneumoniae* strain 2226 cells grown to the midexponential stage were diluted in fresh TSB medium, and 100 μl of the diluted samples was transferred to each well of a 96-well plate. Different concentrations of Dep42 (1 and 10 μg/ml) or SUMO (1 and 10 μg/ml) were added to different wells. The diluted bacterial culture and fresh TSB medium were also included as controls. After incubation at 37°C for 48 h without agitation, the residual biofilm was assessed by CV staining followed by measurement at 595 nm.

The ability of Dep42 to disrupt biofilms of *K. pneumoniae* strain 2226 was also detected. The bacteria were grown in a 96-well plate for 48 h. The supernatant was removed, and attached cells were treated with different concentrations of Dep42 from 1 to 10 μg/ml or SUMO from 1 to 10 μg/ml for 3 h. Biofilm degradation was evaluated by CV staining, and the bacterial load of the treated biofilm was calculated by determining the viable cell count expressed in log_10_.

The minimal inhibitory concentration (MIC) of polymyxin for planktonic cells of *K. pneumoniae* strain 2226 was determined according to the CLSI guidelines (2000), and the MIC for polymyxin was found to be 1 μg/ml. Mature biofilms grown for 48 h were treated with various concentrations of polymyxin (1, 3, 4, 6 μg/ml) for 8 h, and a significant decrease in the bacterial counts was observed at a concentration of 6 μg/ml.

To investigate the effect of combined treatment with Dep42 and antibiotics on biofilm degradation, mature biofilms grown for 48 h were exposed to polymyxin (6 μg/ml) and Dep42. In brief, biofilm formation was induced in 96-well plates for 48 h, and biofilms were washed three times with sterile PBS (1 mM, pH 7.2). Then, the biofilms were divided into five groups: (1) the control group, treated with PBS for 11 h; (2) the SUMO group, treated with SUMO (10 μg/ml) for 11 h; (3) the Dep42 group, treated with Dep42 (10 μg/ml) for 11 h; (4) the polymyxin group, treated with PBS for 3 h and then treated with polymyxin (6 μg/ml) for 8 h; (5) the combination group, treated with Dep42 (10 μg/ml) for 3 h and then treated with polymyxin at a final concentration of 6 μg/ml for 8 h. The biofilms were then scraped, and the bacterial culture was diluted to estimate the viable cell count. The bacterial load of the treated biofilm was calculated by determining the viable cell count expressed in log_10_.

### Statistics

GraphPad Prism (version 5, GraphPad Software, USA) software was used for the statistical analysis. The data were tabulated as mean ± SD. In the bivariate analysis, the *t*-test for independent variables was used to analyze normally distributed data, and the Mann-Whitney U test was used to analyze nonnormally distributed data. In multiple comparisons, one-way ANOVA was used to analyze normally distributed data, and the Kruskal-Wallis test was used to analyze nonnormally distributed data. *p* <0.05 was considered statistically significant.

### Ethics Statement

The strain specimens were collected with the written and informed consent of the patients. The conducts and procedures involved in the present work were approved by the Ethics Committee of Shanghai Jiao Tong University School of Medicine.

## Results

### Capsule Typing of *Klebsiella pneumoniae* Clinical Isolates

All 16 clinically isolated strains were resistant to multiple antibiotics, including meropenem (MEM), imipenem (IPM), cefuroxime (CXM), cefotaxime (CTX), amikacin (AMK), cefazolin (CZO), GEN, and piperacillin (PIP) and were CRKP strains. Based on the *wzi* sequencing data, these 16 *K. pneumoniae* isolates could be grouped into 8 distinct capsular types, namely, K47, K64, K38, K3, K10, K54, K2, and KN2, with 5, 3, 3, 1, 1, 1, 1, and 1 isolates, respectively (Table 1).

### Multilocus Sequence Typing and Pulsed-Field Gel Electrophoresis Typing Analysis

The MLST results indicated that all five K47-type, all three K64-type, and one K38-type isolates belonged to the ST11 type (Table 1). As type K47 and type K64 were the predominant CRKP strains in China (66.1 and 18.3%, respectively) (Liu et al., 2017) and type ST11 was the most prevalent CRKP strain (Qi et al., 2011), it is not surprising that all strains of types K47 and K64 in this study belonged to the ST11 type (Table 1).

The five K47-type isolates and three K64-type isolates were further analyzed by PFGE typing. The results showed that strains with the same capsular type and sequence type (ST) exhibited diverse PFGE patterns (Supplementary Figure 1). The genomic heterogeneity among these strains indicated that the K47-type and K64-type isolates were not from a single clone.

### Morphology and Lytic Spectrum of the Phage SH-KP152226

**Figure 1:**
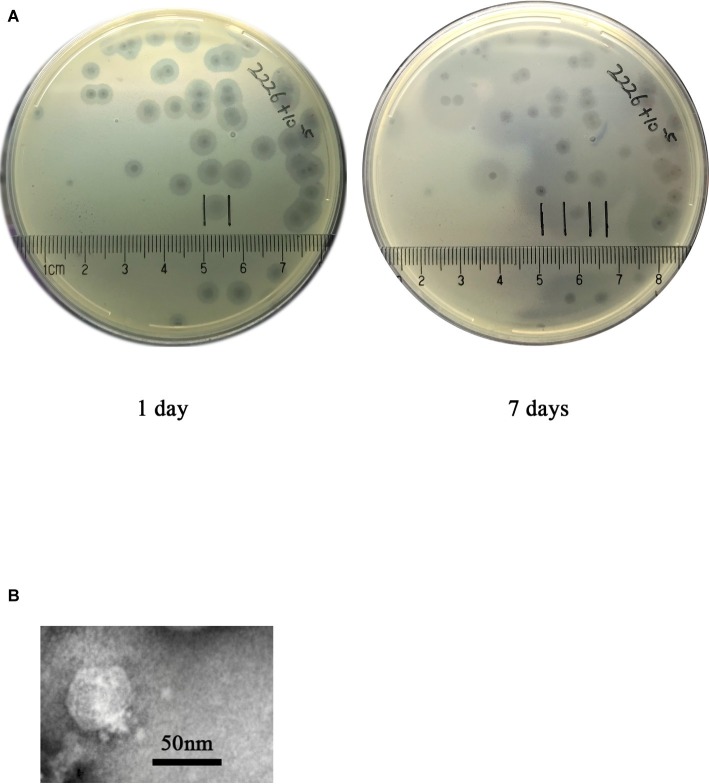
Phage plaque and virion morphology. **(A)** The phage SH-KP152226 produced plaques surrounded by an expanding halo after incubation with *K. pneumoniae* strain 2226. Pure phage plaques were observed on the first day, and the halos surrounding the plaques increased in size after 7 days at 4°C. **(B)** TEM images revealing the morphology of the phage SH-KP152226.

The phage SH-KP152226 was isolated from hospital sewage samples by enrichment with *K. pneumoniae* strain 2226. The plaque morphology after incubation with *K. pneumonia* strain 2226 at 4°C showed clear plaques with halos. The size of the halos was 0.7 cm on day 1 and increased by 1 cm on day 7 (Figure 1A), which suggested the presence of a polysaccharide depolymerase. Using transmission electron microscopy (TEM), we observed that the phage SH-KP152226 possessed a 50-nm head and a 20 nm × 10 nm tail, characteristic of the *Podoviridae* family of phages (Figure 1B).

The lytic spectrum of the phage SH-KP152226 was determined by a spot test on 16 CRKP strains with seven distinct capsular types (Table 1). The results showed that the phage SH-KP152226 produced plaques only on five CRKP strains with capsular type K47 according to *wzi* sequencing (Table 1). After prolonged incubation with type K47 strains, halos surrounding the phage plaques were produced, while other strains developed neither plaques nor halos (Table 1). We also compared the phage infectivity among different host strains. We calculated the EOP value as the ratio of the number of lysis plaques appearing on each susceptible bacterial lawn to the number of plaques on the lawn of the host strain 2226 (Table 1). The results indicated that, except for *K. pneumoniae* strain 1115 (EOP = 0.44), the phage SH-KP152226 had high efficiency toward the tested K47 host strains.

### Genomic Analysis and Classification of the Phage SH-KP152226

**Figure 2:**
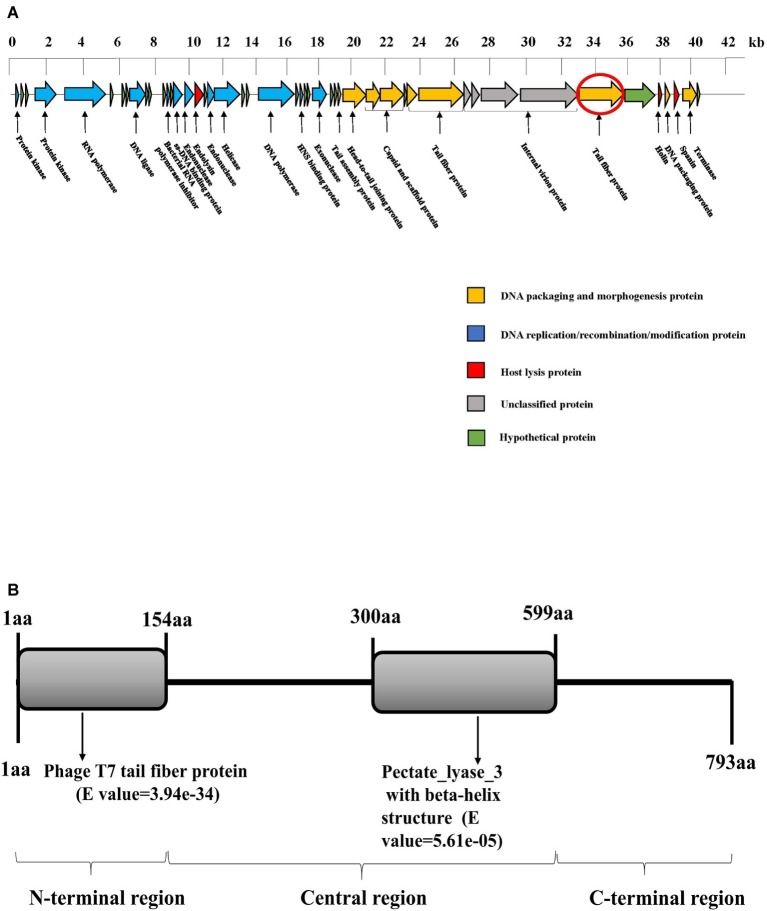
Bioinformatic analysis of the genome of the phage SH-KP152226. **(A)** Gene map of the phage SH-KP152226. Predicted ORFs are shown. The linear map is based on nucleotide sequences of the whole genome. Different color-coded ORFs represent distinct functions: yellow for phage DNA packaging and morphogenesis; blue for phage DNA replication, recombination, and modification; red for host lysis; gray for unclassified functions; and green for hypothetical proteins. The red circle denotes ORF42, the gene encoding the putative polysaccharide depolymerase. **(B)** Bioinformatic analysis of the putative depolymerase of the phage SH-KP152226. Based on *in silico* analysis using integrated information from BLASTp and HHpred, the result depicts a 793-aa protein with two conserved region structures: phage T7 tail fiber protein (residue 1~154) and pectate lyase 3 with a beta-helix structure (residue 300~599).

Whole-genome sequencing demonstrated that SH-KP152226 has a linear double-stranded genome of 41,420 bp, with a GC content of 52.70%. The genomic sequence of phage SH-KP152226 was deposited in the NCBI GenBank database (accession number: MK903728). This sequence encodes 48 predicted proteins (Figure 2A). The average size of these genes was 766 bp. The BLAST results showed that there are two phages with high homology to SH-KP152226: *Klebsiella* phage vB_KpnP_IME205 (accession number: KU183006, query cover = 93.00%, identity = 94.86%) and *Klebsiella* phage IME304 (accession number: MK795385, query cover = 92.00%, identity = 93.99%). The tape measure protein, which is responsible for tail assembly in the *Myoviridae* and *Siphoviridae* families (Garcia-Heredia et al., 2013), was not found in the genome of the phage SH-KP2226, indicating that this phage belongs to the *Podoviridae* family.

The phage SH-KP2226 is further classified into the *Autographivirinae* subfamily due to the existence of a single-subunit RNA polymerase gene (ORF5), which has been reported to function as a self-transcribing gene (Eriksson et al., 2015). Twenty-eight proteins with distinctive functions are encoded by the phage SH-KP152226 and can be divided into four categories (Figure 2B): DNA assembly and morphologically related proteins (9 proteins); DNA replication, recombination, and modification proteins (12 proteins); host lysis proteins (3 proteins); and unclassified proteins (4 proteins). There are no virulent or drug-resistant genes based on the genetic analysis of the phage SH-KP15226 against the VFDB and ARDB.

Phage-mediated lysis is initiated when holin proteins form holes in the inner membrane of the host bacteria, followed by the release of endolysin into the periplasm to degrade the peptidoglycan in the bacterial cell wall (Cahill and Young, 2019). Genes encoding this holin-endolysin system are usually in close arrangement in the phage genome (Oakley et al., 2011; Cahill and Young, 2019). However, genes encoding the holin (ORF44) and the endolysin (ORF17) are distant from each other in the genome of SH-KP152226. The distant arrangement of the holin-endolysin system was also reported in a few other phages, such as *Klebsiella* phage vB_KpnP_KpV767 (accession number: KX712070) (Solovieva et al., 2018). Spanins are another type of phage lysis protein required to disrupt the outer membrane. They are classified into two components (i-spanin and o-spanin) and a unimolecular component (Kongari et al., 2018). In the phage SH-KP15226, the gene product annotated as i-spanin (ORF46), but not o-spanin, was found. The phage vB_KpnP_IME205, which is the closest homolog to the phage SH-KP152226, also encodes only the i-spanin. The unique features of the holin-endolysin system and spanins of the phage SH-KP152226 should be further explored in the future.

### Analysis of Predicted Genes Encoding a Polysaccharide Depolymerase

Expansion of the translucent halos surrounding the plaques was clearly observed in the experiment, indicating that phage SH-KP152226 has polysaccharide depolymerase activity. Phage depolymerase is often found in the tail fiber protein or the tail spike protein. Accordingly, we hypothesized and demonstrated that ORF42, a putative tail fiber protein, possesses polysaccharide depolymerase activity. There are three regions in the ORF42 protein (Figure 2B), namely, the N-terminal region, central region, and C-terminal region. The amino acid sequence of the N-terminal region (residues 1~154) shares approximately 32.09–75.69% identity with that of T7-like phages; this region is responsible for the flexible connection to the tail structure or baseplate. In the central region (residue 300~599), a pectate pectate_lyase_3 with the beta-helix structure was found. This structure functions by binding to and degrading the polysaccharide component of the bacterium and is essential for initiation of phage infection. No significant similarity was observed in the C-terminal region, which might be involved in protein trimerization or binding to the inner component (host receptor) of the bacterium. Thus, the gene product of ORF42 is a putative novel polysaccharide depolymerase from the phage of *K. pneumoniae*.

### Cloning and Expression of the Depolymerase

**Figure 3:**
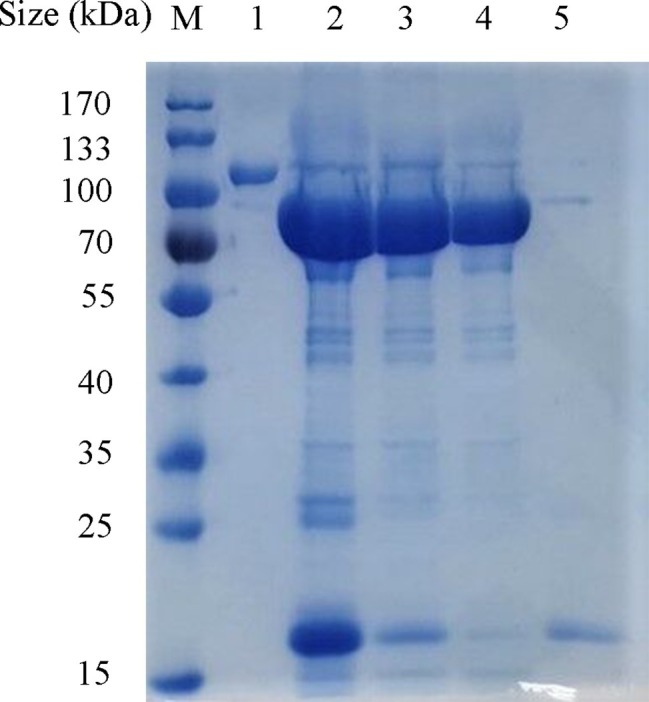
Expression and purification of recombinant Dep42. The proteins from different purification stages were separated by 10% SDS-PAGE, along with the PageRuler prestained protein ladder. Lane M, protein marker; lane 1, purified Dep42-SUMO fusion protein; lane 2, Dep42-SUMO fusion protein treated with SUMO protease; lanes 3 and 4, recombinant Dep42 after removal of the SUMO tag with the samples reapplied to the Ni-NTA column once and twice, respectively; lane 5, cleaved SUMO tag eluted from the Ni-NTA column with 300 mM imidazole.

The ORF42 gene of the phage SH-KP152226 was cloned into an expression plasmid, and the recombinant protein was expressed in *E. coli* BL21 cells. Ni-NTA chromatography was employed to purify the tagged protein. The purified recombinant protein Dep42 migrated as a single band with an approximate MW of 102 kDa on an SDS-PAGE gel (Figure 3). The SUMO-Dep42 protein was then treated with SUMO protease and reapplied to the Ni-NTA column to remove the cleaved His-tagged SUMO and the uncleaved residual fusion protein. Finally, purified Dep42 (approximately 85 kDa) was confirmed by SDS-PAGE to have a purity of more than 90% (Figure 3).

### Degradation of Extracellular Polysaccharide by the Recombinant Depolymerase

**Figure 4:**
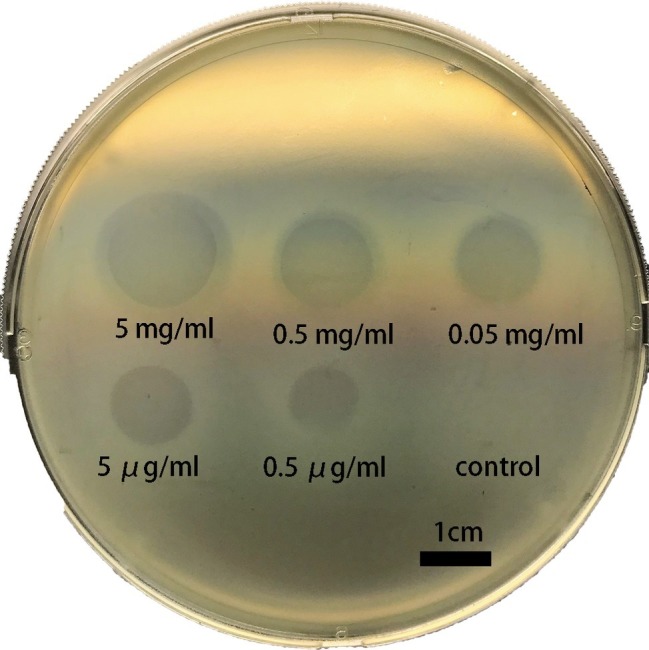
Dep42 activity against *K. pneumoniae* strain 2226 on a plate. Different concentrations of Dep42 (ranging from 5 mg/ml to 0.5 μg/ml) were applied to a plate containing *K. pneumoniae* strain 2226. SUMO (5 mg/ml) was used as a control.

The degradation activity of Dep42 was determined with the spot test by measuring the halo zones against *K. pneumoniae* strain 2226 (Figure 4). The results indicated that Dep42 was active against *K. pneumoniae* strain 2226, and the halo zone was detected at concentrations as low as 0.5 μg/ml.

The specificity of Dep42 was examined on 16 *K. pneumoniae* strains using the spot test. The phage SH-KP152226 was used as a positive control. We found that only type K47 of *K. pneumoniae* was susceptible to Dep42 treatment, consistent with the observation for the phage SH-KP152226 (Table 1).

**Figure 5:**
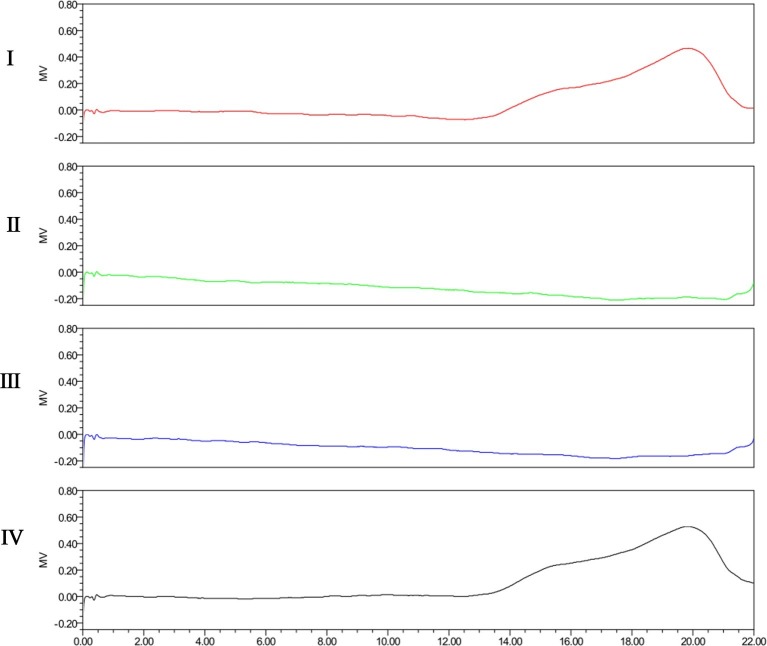
SEC-HPLC analysis of EPS treated with Dep42. I, purified untreated EPS; II, EPS treated with 10 μg/ml Dep42 at 37°C; III, EPS treated with 10 μg/ml Dep42 at 25°C; IV, EPS incubated with 10 μg/ml SUMO at 37°C.

The degradation activity of Dep42 was further confirmed on the purified EPS from *K. pneumoniae* strain 2226. As shown in Figure 5, the SEC-HPLC analysis of untreated EPS solution showed a main peak with a retention time from 18 to 20 min. After incubation with Dep42 (10 μg/ml) at 37°C for 30 min, the peak signal of EPS disappeared from 18 to 20 min, suggesting that EPS was degraded by Dep42. In the control, SUMO showed no EPS-degrading activity (Figure 5). These results demonstrated that EPS from *K. pneumoniae* strain 2226 can be degraded by Dep42.

### Characterization of the Depolymerase Activity

**Figure 6:**
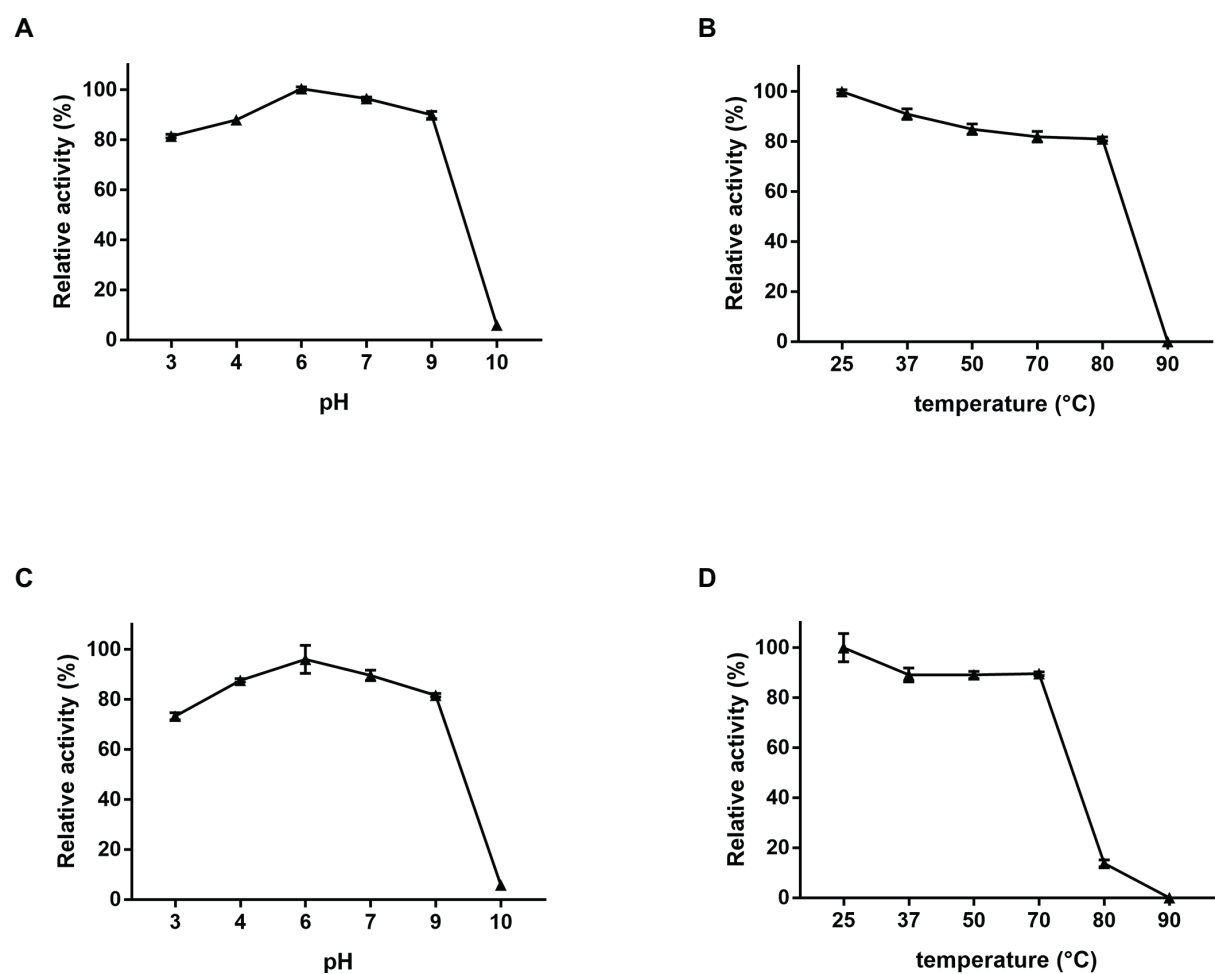
EPS-degrading activity of Dep42. **(A)** The effect of pH on the enzyme activity at 25°C. The following pH buffer systems were used: 50 mM sodium acetate buffer (pH 3.0–5.0), 50 mM Na_2_HPO_4_ (pH 6.0–7.0), 50 mM Tris-HCl buffer (pH 8.0–9.0), and 100 mM NaHCO_3_ (pH 10.0–11.0). **(B)** The effect of different temperatures on the enzyme activity at pH 6.0. **(C)** Stability and activity of the enzyme under different pH buffers. Dep42 was preincubated in different pH buffers for 30 min, and the residual activity was measured at 25°C. **(D)** Effect of temperature on enzyme stability and activity. Dep42 was preincubated at different temperatures for 30 min, and the residual activity was measured at pH 6.0. Relative enzyme activity was presented as a percentage of the highest activity value. Error bars represent the standard error of the mean.

The optimal activity and stability of Dep42 was further studied with regard to pH and temperature. The degradation activity was determined by assaying the generation of reducing sugars following the enzyme treatment. The relative enzymatic activity was presented as a percentage of the highest activity value observed at pH 6.0 and 25°C (Figure 6). Figure 6A shows that this recombinant protein is highly active from pH 3.0 to pH 9.0, with relative activities ranging from 81.4 ± 0.50 to 100.5 ± 0.38%, respectively. Figure 6B shows that the protein retains high activity from 25 to 80°C, with relative activity ranging from 100.0 ± 0.51 to 80.5 ± 0.56%, respectively.

These data also demonstrated that recombinant Dep42 maintained high stability and activity under extreme conditions. For instance, when preincubated at 70°C for 30 min, Dep42 had an enzymatic activity of 89.6 ± 0.5%. When it was preincubated at pH 3.0 or pH 9.0, the enzymatic activity was 73.2 ± 0.99% or 81.7 ± 0.50%, respectively (Figures 6C,D).

### Antibiofilm Activity of the Depolymerase

**Figure 7:**
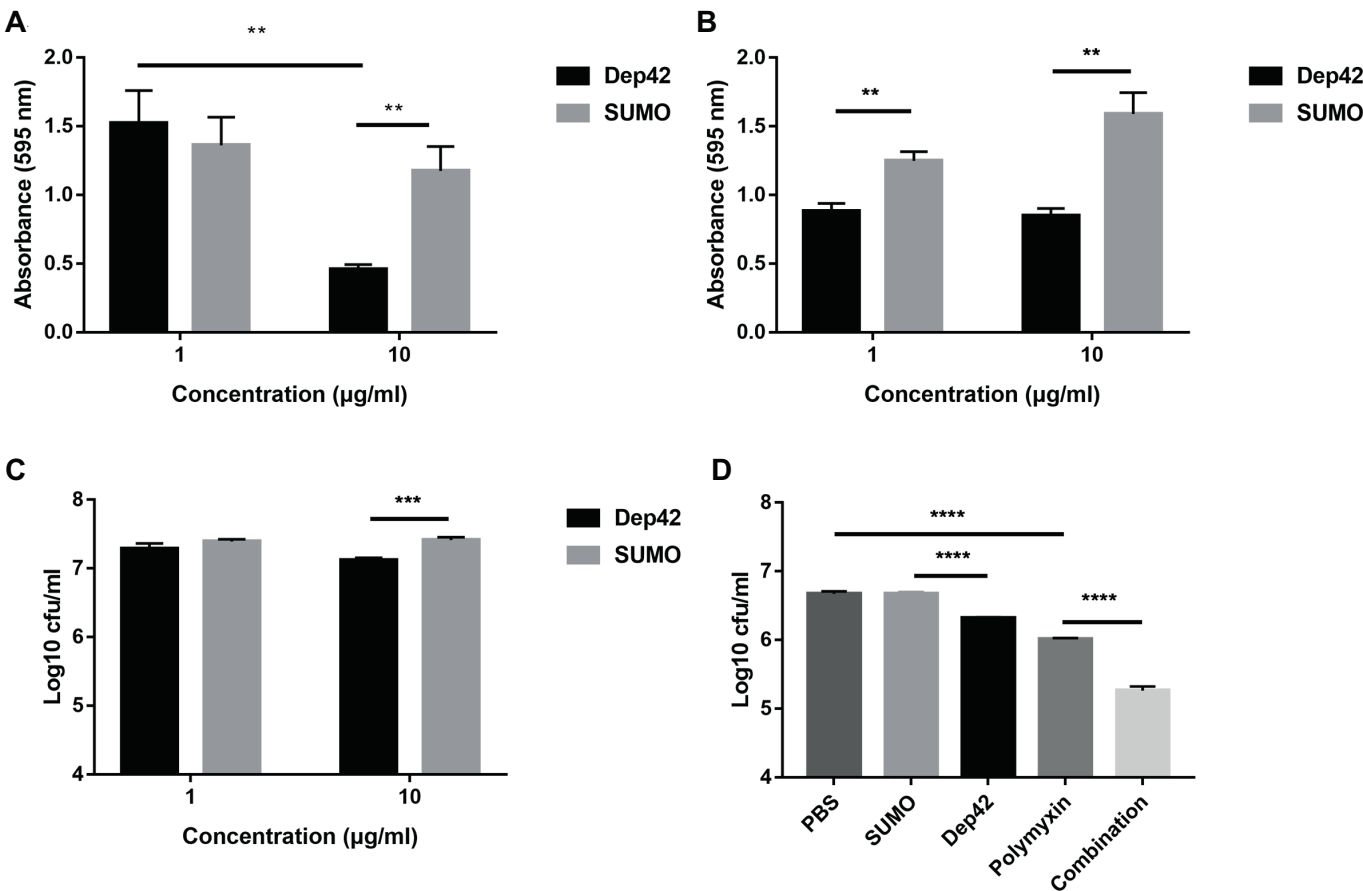
Antibiofilm and antibacterial activity of Dep42. **(A)** Dep42 inhibited the formation of biofilms. Dep42 or SUMO at 1 and 10 μg/ml was incubated with *K. pneumoniae* 2226 in 96-well plates for 48 h. The residual biofilm was assessed by crystal violet staining, and the absorbance was measured at 595 nm. **(B,C)** Dep42 disrupted the mature biofilm. Biofilm formation of *K. pneumoniae* strain 2226 was induced in 96-well plates for 48 h, and then, the biofilm was treated with depolymerase (black bar) or SUMO (gray bars) at 1 and 10 μg/ml for 3 h. The residual biofilm was assessed by crystal violet staining **(B)** and viable cell counting **(C)**. **(D)** Dep42 enhanced the antimicrobial activity of polymyxin. Biofilm formation of *K. pneumoniae* strain 2226 was induced in 96-well plates for 48 h, and then, the biofilm was treated with Dep42 (10 μg/ml), polymyxin (6 μg/ml), or Dep42 (10 μg/ml) followed by polymyxin (6 μg/ml) as indicated. The treatment groups with PBS and SUMO (10 μg/ml) were included as negative controls. The viable bacterial count was determined on LB agar plates. The data are shown as the mean ± SEM; * indicates *p* < 0.05, **p < 0.01, and ***p < 0.001.

The effect of Dep42 on biofilm formation by *K. pneumoniae* strain 2226 was assessed using 96-well plates. To evaluate the ability of Dep42 to inhibit biofilm formation, different concentrations (1 and 10 μg/ml) of Dep42 were incubated with *K. pneumoniae* strain 2226 for 48 h. The results showed that the absorbance value of the Dep42-treated group (10 μg/ml) decreased significantly to 0.717 ± 0.15 compared with that of the control group (Figure 7A). This result suggested that Dep42 could prevent biofilm formation.

The ability of Dep42 to degrade biofilms was also assessed using mature biofilms (48 h old). According to the results of the CV staining assay, the absorbance value of the total biomass that remained attached after 1 or 10 μg/ml Dep42 treatment was reduced by 0.365 ± 0.06 or 0.743 ± 0.09, respectively, compared with that of the control (Figure 7B). The data for the direct viable counts showed that the average bacterial counts in the biofilm were 7.28 ± 0.12 log and 7.39 ± 0.051 log after treatment with SUMO (1 μg/ml) and Dep42 (1 μg/ml), respectively, and no significant difference was observed between the two groups. Treatment with 10 μg/ml Dep42 resulted in a reduction of 0.290 ± 0.05 log in the bacterial counts of the biofilm (*p* < 0.001) compared to the control group (Figure 7C). These data suggested that Dep42 can not only inhibit biofilm formation but also degrade the formed biofilm.

### Enhanced Antimicrobial Activity of Polymyxin

The high activity of Dep42 in degrading mature biofilms of *K. pneumoniae* led us to investigate the efficacy of combined treatment with Dep42 and antibiotics to determine if there was a synergistic effect. The results showed that the average bacterial count of the polymyxin group was 6.013 ± 0.125 log, which was 0.660 ± 0.036 log lower than that of the PBS group (Figure 7D). The average bacterial count for the Dep42 group was 6.317 ± 0.01 log, which was 0.356 ± 0.09 log lower than that for the SUMO group (*p* < 0.05) (Figure 7D). Interestingly, the average bacterial count for the combined group was 5.260 ± 0.05 log, exhibiting a significant reduction of 0.743 ± 0.05 log compared with the bacterial count of the polymyxin group (Figure 7D). As a control, the SUMO group showed no difference in the bacterial count compared to the PBS group. These data strongly indicated that Dep42 has a synergistic effect in enhancing the efficacy of polymyxin through the degradation of *K. pneumoniae* biofilms.

## Discussion

Biofilm formation plays an important role in the ability of microbial organisms to evade host defense systems and is thus considered a leading pathogenic factor in the clinical setting (Pinna et al., 2005; Vuotto et al., 2017). Biofilms have been linked to many clinical pathogenic conditions, such as urinary tract infection, chronic respiratory infection, and chronic otitis media (Hatt and Rather, 2008; Childers et al., 2013). Therapeutic intervention for bacterial infections involving biofilm formation has been extremely challenging, especially for multidrug-resistant infections. Therefore, there is an urgent and unmet medical need to develop novel therapies to combat the ever-increasing infections.

*K. pneumoniae* is a clinically important bacterial pathogen that is most commonly isolated from biofilms (Donlan, 2001; Donlan and Costerton, 2002; Parasion et al., 2014). Recently, the development of alternative approaches to treat *K. pneumoniae* infections has gained increasing attention due to the high prevalence of CRKP and the frequent failure of antibiotic therapy. The application of phage depolymerases appears to be one of the promising treatment strategies for *K. pneumoniae* infection (Lin et al., 2014; Pan et al., 2019; Wang et al., 2019).

To date, 23 polysaccharide depolymerases (experimentally confirmed) encoded by *Klebsiella* phages have been identified, and some of these depolymerases showed activity against *K. pneumoniae* infection (Latka et al., 2017; Pan et al., 2019). The previously reported depolymerases were identified for 20 *Klebsiella* capsular types, namely, K1, K2, K3, K5, K8, K11, K13, K21, K25, K30, K35, K56, K63, K64, K69, KN1, KN2, KN3, KN4, and KN5 (Pan et al., 2019). However, the depolymerase targeting type K47 *K. pneumoniae* has not yet been identified.

ST11 is the dominant CRKP clone in China, accounting for up to 60% of the clinical CRKP strains (Zhang et al., 2017). An epidemiological study found that the predominant capsular type of CRKP strains in China is the K47 serotype, accounting for 66.1% (Liu et al., 2017). Recently, Danxia Gu and his colleagues reported a fatal outbreak caused by CRKP isolates in a hospital in China (Gu et al., 2018). All the strains in that study were found to be type ST11 and the K47 serotype (Gu et al., 2018). The prevalence of K47 CRKP is increasingly becoming a serious threat to public health in China. In this study, we identified a novel depolymerase, Dep42, from a *Klebsiella* phage and determined that it could selectively recognize and degrade the capsule of the K47 serotype. The degradation activity of Dep42 makes it a very attractive agent for the control of K47 CRKP infections in China.

It is well documented that tail fiber proteins or tail spike proteins of phages usually possess depolymerase activity for polysaccharides (Stirm et al., 1971; Chaturongakul and Ounjai, 2014). In this study, ORF42 of the phage SH-KP152226, annotated as the tail fiber protein gene, was subjected to BLAST analysis. The BLASTp alignment results showed that the tail fiber protein encoded by the *Klebsiella* phage vB_KpnP_IME205 (accession number: ALT58497) shared the highest homology (identity = 98.36%, query cover = 100%) with the protein encoded by ORF42 of phage SH-KP152226. However, the polysaccharide depolymerase protein encoded by the phage vB_KpnP_IME205 has not been reported with experimental validation. The experimentally confirmed polysaccharide depolymerases encoded by *Klebsiella* phages (Supplementary Table) include 10 depolymerases containing the pectate lyase region. According to the BLASTp result, the amino sequence of Dep42 shares similarity with these 10 depolymerases. Among these depolymerases, the experimentally confirmed depolymerase S2-6 (accession number: WP_020801644) encoded by *Klebsiella* phage K64-1 (accession number: LC121097) shares the highest homology with the gene product of ORF42 (identity = 43.90%, query cover = 36%). Furthermore, the alignment result of different regions between ORF42 of the phage SH-KP152226 and the gene encoding depolymerase S2-6 indicates the differences between these sequences in detail. The central regions (pectate lyase with a beta-helix) of these two genes share some similarity (query cover = 62%, identity = 51.85%), the N-terminal regions share less similarity (query cover = 8%, identity = 28.00%), and the C-terminal regions share no similarity, which might explain the specificity of the depolymerase for the particular bacterial serotype (S2-6 for capsular types K30 and K69, Dep42 for capsular type K47). Here, we first identified the depolymerase Dep42, which specifically targets the type K47 capsule of *K. pneumoniae*. We speculate that Dep42 may represent a potential candidate either as a valuable tool for the characterization of clinical strains or as a promising molecular identity for future therapeutic development.

The depolymerization activity of Dep42 was investigated under a wide range of environmental conditions using EPS from the K47 strain. This protein showed robust enzymatic activities and stability even under extreme conditions. Similar to most phage depolymerases, such as Dp42 (Wang et al., 2019), depoKP36 (Majkowska-Skrobek et al., 2016), and po48 (Liu et al., 2019), Dep42 remained highly active under a wide range of pH values and temperatures. The activity of Dep42 was optimal from pH 3 to pH 9 and at temperatures from 25 to 70°C, which indicates that the protein will have maximal efficacy during therapy. For example, it might be used for the treatment of urinary tract infection, which typically has a pH from 4 to 8 (Yang et al., 2014). From this perspective, Dep42 represents an attractive candidate serving as the anti-*K. pneumoniae* agent in an obstacle approach for catheter reservation by combination with traditional antimicrobial agents.

Bacterial biofilms are communities of bacterial cells surrounded by a protective extracellular matrix composed of proteins, nucleic acids, polysaccharides, and lipids (Flemming et al., 2007; Flemming and Wingender, 2010). Bacterial pathogens in biofilms are highly tolerant to antibiotic treatment and to the host immune system (Lewis, 2001; Yang et al., 2011; Yan and Bassler, 2019).

There is a growing need for the development of new strategies to control bacterial infections associated with biofilms. The discovery of depolymerases capable of degrading EPS offers a novel approach for the treatment of infections. More recently, several works found that depolymerases alone or in combination with antibiotics can efficiently prevent or eradicate biofilm-associated infections. Alkawash and colleagues reported that the biofilm infection caused by mucoid *Pseudomonas aeruginosa* could not be eradicated by GEN alone. However, combination of GEN with depolymerase could effectively eliminate mucoid *P. aeruginosa* in biofilms (Alkawash et al., 2006). Another research group found that the combined use of depolymerase and GEN led to a significant reduction in the counts of *K. pneumoniae* in biofilms, while GEN alone did not show any efficacy (Bansal et al., 2015). In this study, we first showed that the recombinant depolymerase Dep42 was able to significantly inhibit biofilm formation and degrade mature biofilms. Our data demonstrated that Dep42 had a synergistic effect on the antibiotic activity against *K. pneumoniae* in biofilms when the polymerase was combined with polymyxin. These data are consistent with previous findings that showed that combined treatment using ciprofloxacin and a depolymerase worked effectively against mature biofilms (Verma et al., 2010).

In this study, the total biomass of mature biofilm treated with 1 μg/ml Dep42 was reduced, but the bacterial count remained unchanged (Figures 7B,C). This was possibly because CV staining reflects the total biomass of biofilms, including total bacteria, proteins, polysaccharides, and nucleic acids, while the CFU count represents the number of living bacteria. Destruction of biofilm polysaccharide components after treatment with depolymerase at a low dose leads to reduced biofilm stability, which may result in the release of bacteria from the biofilm or may simply cause a decrease in biofilm composition without changing the number of living bacteria.

The role of biofilm cell dispersal in the pathogenesis of infections is not well understood. A previous study showed that cells released from biofilms were highly virulent and could potentially cause bacterial dissemination or septic shock (Wang et al., 2011; Chua et al., 2014; França et al., 2016). It is important to note that Dep42 in this study is not bactericidal. Although Dep42 treatment led to the generation of “capsule-stripped” bacteria, which are presumably much easier to eradicate by antibiotics and the host immune system, to degrade biofilms, a combination of Dep42 and antimicrobial agents should be considered to eliminate dispersed cells (Stewart and Costerton, 2001; Mushtaq et al., 2005; Zelmer et al., 2010; Bansal et al., 2014, 2015).

In conclusion, we identified a novel depolymerase and characterized its catalytic features. Our findings showed the high degradation activity of Dep42 for the exopolysaccharide and demonstrated the synergistic effect of the combined use of Dep42 and polymyxin against type K47 *K. pneumoniae*. All the data suggest that Dep42 is potentially a promising candidate for controlling CRKP infections and *K. pneumoniae* infections in combination with antibiotics.

## Accession Number

The annotated genomic sequence of the phage SH-KP152226 was deposited in the GenBank database at NCBI under the accession number MK903728.

## Data Availability Statement

The datasets generated for this study can be found in the MK903728.

## Author Contributions

QL, PH, and YW drafted the main manuscript and performed the data analysis. QL, PH, YW, RW, MX, YL, XZ, QL and JQ planned and performed experiments. QL, PH, YW, and RW were responsible for experimental design. All authors revised the manuscript.

### Conflict of Interest

XZ is an employer of Shanghai Ruizhou Biotech Co. Ltd.

The remaining authors declare that the research was conducted in the absence of any commercial or financial relationships that could be construed as a potential conflict of interest.

## Footnotes

^1^https://bigsdb.pasteur.fr/klebsiella/primers_used.html

^2^https://blast.ncbi.nlm.nih.gov/Blast.cgi

## Supplementary Material

SUPPLEMENTARY FIGURE S1 Genotyping of *K. pneumoniae* strains by pulsed-field gel electrophoresis (PFGE). Genomic DNA of *K. pneumoniae* strains digested with *Xba*I was separated by PFGE. Lane 1, strain 1093; lane 2, strain 1115; lane 3, strain 2226; lane 4, strain 2302; lane 5, strain 2340; lane 6, strain 1044; lane 7, strain 2450; lane 8, strain 2482. Band sizes are in kb.
