# Supplementary figures and images for: A Novel Polysaccharide Depolymerase Encoded by the Phage SH-KP152226 Confers Specific Activity Against Multidrug-Resistant Klebsiella pneumoniae via Biofilm Degradation
Source: Front Microbiol. 2019 Dec 3;10:2768. doi: 10.3389/fmicb.2019.02768 (PMC6901502; doi:10.3389/fmicb.2019.02768)

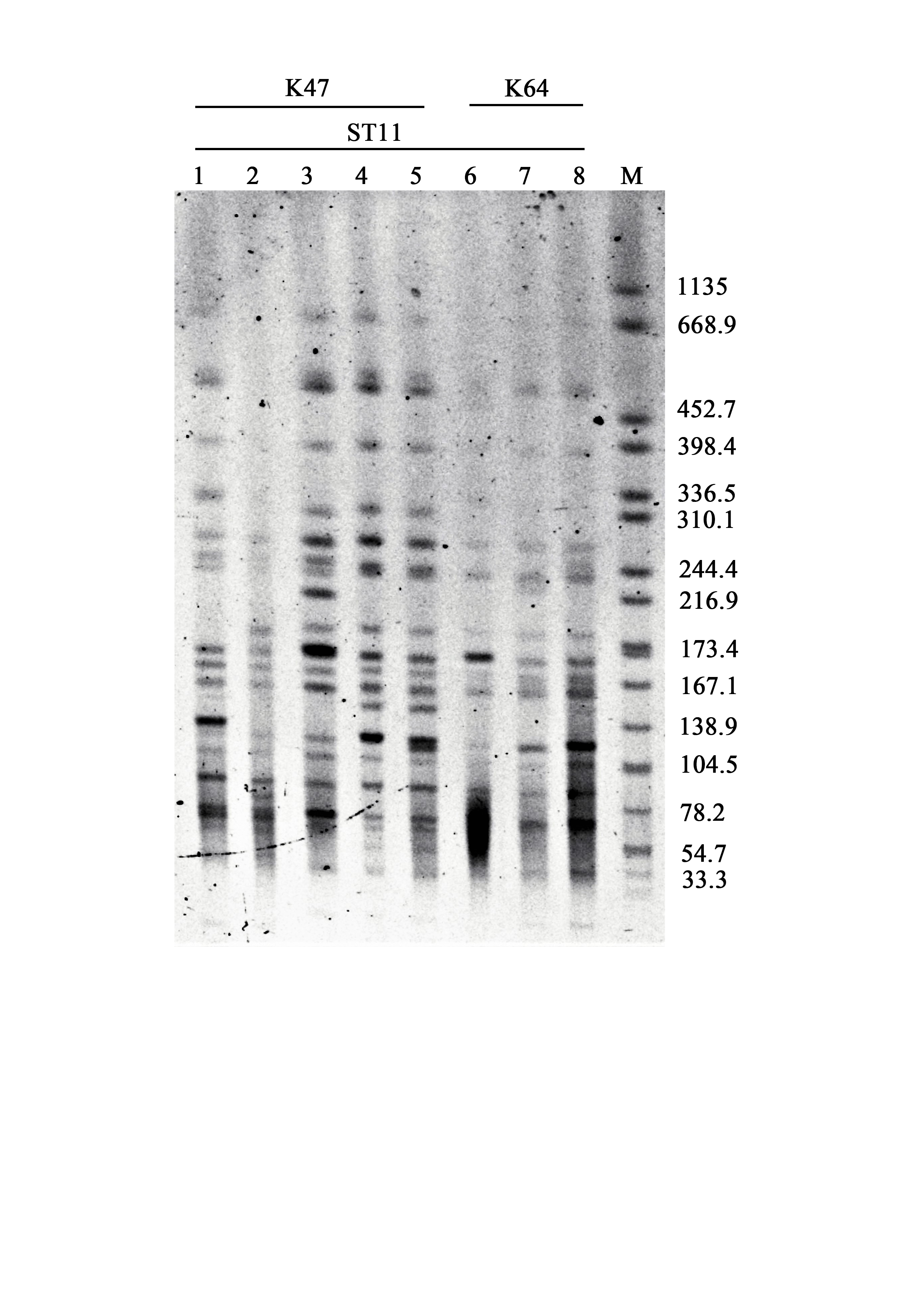

Supplement: SUPPLEMENTARY FIGURE S1 — Genotyping of K. pneumoniae strains by pulsed-field gel electrophoresis (PFGE). Genomic DNA of K. pneumoniae strains digested with XbaI was separated by PFGE. Lane 1, strain 1093; lane 2, strain 1115; lane 3, strain 2226; lane 4, strain 2302; lane 5, strain 2340; lane 6, strain 1044; lane 7, strain 2450; lane 8, strain 2482. Band sizes are in kb. [file Image_1.TIF]
