# Supplementary material for: A Novel Polysaccharide Depolymerase Encoded by the Phage SH-KP152226 Confers Specific Activity Against Multidrug-Resistant Klebsiella pneumoniae via Biofilm Degradation
Source: Front Microbiol. 2019 Dec 3;10:2768. doi: 10.3389/fmicb.2019.02768 (PMC6901502; doi:10.3389/fmicb.2019.02768)
Supplement: Supplementary file 2 [file Table_1.DOCX]

Supplement Table Identified *Klebsiella* phages and their polysaccharide depolymerases.

| **Phage** | **Phage genome accession no.** | **Host** | **Capsule depolymerase** | **Capsule depolymerase accession no.** | **Capsule depolymerase activity** | **Pectate-degrading ability** | **Similarity compared with Dep42 gene of phage SH-KP152226** | **Reference** |
| --- | --- | --- | --- | --- | --- | --- | --- | --- |
| KN1-1 | LC413193 | KN1 | KN1dep | BBF66844 | KN1 |  | N.S. | ([Pan et al., 2019](#_ENREF_7)) |
| KN3-1 | LC413194 | KN3 | KN3dep | BBF66867 | KN3 | Yes | N.S. |  |
|  |  |  | K56dep | BBF66868 | K56 |  | N.S. |  |
| KN4-1 | LC413195 | KN4 | KN4dep | BBF66888 | KN4 |  | N.S. |  |
| K64-1 | LC121097 | K1, K11, K21, K25, K30, K35, K64, K69, KN4, KN5 | S1-1 | YP_002003830 | K11 |  | N.S. | ([Pan et al., 2017](#_ENREF_6)) |
|  |  |  | S1-2 | YP_008532048 | KN4 |  | N.S. |  |
|  |  |  | S1-3 | AGF88658 | K21 |  | N.S. |  |
|  |  |  | S2-1 | YP_007010682 | KN5 |  | N.S. |  |
|  |  |  | S2-2 | WP_020326882 | K25 |  | N.S. |  |
|  |  |  | S2-3 | YP_654147 | K35 |  | N.S. |  |
|  |  |  | S2-4 | YP_007003187 | K1 |  | N.S. |  |
|  |  |  | S2-5 | YP_398994 | K64 |  | N.S. |  |
|  |  |  | S2-6 | WP_020801644 | K30/K69 | Yes | 43.90% |  |
| KpV71 | KU666550 | K1, K62 | kpv71_52 | AMQ66478 | K1/K62 | Yes | 38.71% | ([Solovieva et al., 2018](#_ENREF_8)) |
| KpV74 | KY385423 | K2, K13 | kpv74_56 | APZ82768 | K2/K13 | Yes | 50.00% |  |
| K5-2 | KY389315 | K5, K30, K69 | K5-2 ORF37 | APZ82804 | K5/K30/K69 | Yes | N.S. | ([Hsieh et al., 2017](#_ENREF_1)) |
| K5-4 | KY389316 | K5, K8 | K5-4 ORF37 | APZ82847 | K8 | Yes | N.S. |  |
|  |  |  | K5-4 ORF38 | APZ82848 | K5 | Yes | N.S. |  |
| KP36 | NC_029099 | K63 | depoKP36 | YP_009226010 | K63 | Yes | 30.77% | ([Majkowska-Skrobek et al., 2016](#_ENREF_5)) |
| 0507-KN2-1 | AB797215 | KN2 | ORF96 | BAN78446 | KN2 |  | N.S. | ([Hsu et al., 2013](#_ENREF_2)) |
| KP32 | NC_013647 | K3, K21 | KP32gp37 | YP_003347555 | K3 | Yes | N.S. | ([Majkowska-Skrobek et al., 2018](#_ENREF_4)) |
|  |  |  | KP32gp38 | YP_003347556 | K21 |  | N.S. |  |
| NTUH-K2044-K1-1 | NC_025418 | K1 | K1-ORF34 | YP_009098385 | K1 | Yes | 38.71% | ([Lin et al., 2014](#_ENREF_3)) |

N.S., no significant similarity.

Hsieh, P.F., Lin, H.H., Lin, T.L., Chen, Y.Y., and Wang, J.T. (2017) Two T7-like Bacteriophages, K5-2 and K5-4, Each Encodes Two Capsule Depolymerases: Isolation and Functional Characterization. *Sci Rep* **7**: 4624.

Hsu, C.R., Lin, T.L., Pan, Y.J., Hsieh, P.F., and Wang, J.T. (2013) Isolation of a bacteriophage specific for a new capsular type of Klebsiella pneumoniae and characterization of its polysaccharide depolymerase. *PLoS One* **8**: e70092.

Lin, T.L., Hsieh, P.F., Huang, Y.T., Lee, W.C., Tsai, Y.T., Su, P.A. et al. (2014) Isolation of a bacteriophage and its depolymerase specific for K1 capsule of Klebsiella pneumoniae: implication in typing and treatment. *J Infect Dis* **210**: 1734-1744.

Majkowska-Skrobek, G., Latka, A., Berisio, R., Squeglia, F., Maciejewska, B., Briers, Y., and Drulis-Kawa, Z. (2018) Phage-Borne Depolymerases Decrease Klebsiella pneumoniae Resistance to Innate Defense Mechanisms. *Front Microbiol* **9**: 2517.

Majkowska-Skrobek, G., Latka, A., Berisio, R., Maciejewska, B., Squeglia, F., Romano, M. et al. (2016) Capsule-Targeting Depolymerase, Derived from Klebsiella KP36 Phage, as a Tool for the Development of Anti-Virulent Strategy. *Viruses* **8**.

Pan, Y.J., Lin, T.L., Chen, C.C., Tsai, Y.T., Cheng, Y.H., Chen, Y.Y. et al. (2017) Klebsiella Phage PhiK64-1 Encodes Multiple Depolymerases for Multiple Host Capsular Types. *J Virol* **91**.

Pan, Y.J., Lin, T.L., Chen, Y.Y., Lai, P.H., Tsai, Y.T., Hsu, C.R. et al. (2019) Identification of three podoviruses infecting Klebsiella encoding capsule depolymerases that digest specific capsular types. *Microb Biotechnol* **12**: 472-486.

Solovieva, E.V., Myakinina, V.P., Kislichkina, A.A., Krasilnikova, V.M., Verevkin, V.V., Mochalov, V.V. et al. (2018) Comparative genome analysis of novel Podoviruses lytic for hypermucoviscous Klebsiella pneumoniae of K1, K2, and K57 capsular types. *Virus Res* **243**: 10-18.
